# Mechanisms of traditional Chinese medicine regulating polycystic ovary syndrome through gut microbiota: a review

**DOI:** 10.3389/fendo.2025.1610869

**Published:** 2025-08-06

**Authors:** Xiaomei Jiang, Yongfeng Wang, Yu Hua, Hong Tang, Heyue Li

**Affiliations:** Department of Gynecology, Seventh People’s Hospital of Shanghai University of Traditional Chinese Medicine, Shanghai, China

**Keywords:** polycystic ovary syndrome, traditional Chinese medicine, gut microbiota, mechanisms, hyperandrogenism, insulin resistance, inflammation

## Abstract

Polycystic ovary syndrome (PCOS) is a prevalent endocrine disorder that adversely affects women’s reproductive and metabolic health conditions. Recent studies have highlighted the significant contributory role of gut microbiota in the pathogenesis of PCOS, indicating a complex interplay between the microbiome and the syndrome’s clinical manifestations. Given this connection, Traditional Chinese Medicine (TCM), a well-established therapeutic approach, has demonstrated potential efficacy in modulating gut microbiota and alleviating symptoms associated with PCOS. This review aims to summarize and analyze current research on the effects of TCM on gut microbiota in individuals with PCOS, exploring underlying mechanisms and relevant findings to provide insights for future clinical applications and improve understanding of TCM’s role in managing PCOS.

## 1 Introduction

Polycystic ovary syndrome (PCOS) is one of the most common endocrine disorders in women of reproductive age, affecting 4-20% of this population worldwide (1, 2). PCOS can lead to ovulation disorders resulting in infertility. It is characterized by chronic ovulation disorders, hyperandrogenemia, and bilateral polycystic ovaries, often accompanied by insulin resistance, menstrual irregularities, hirsutism, acne, obesity, and other metabolic conditions (3). These symptoms not only impact the patient’s fertility but also pose a threat to their quality of life and long-term health. Currently, the pathogenesis of PCOS remains partially understood. Previous studies have indicated that genetic, neuroendocrine, and lifestyle factors play important roles in the pathophysiology of PCOS (4). Some studies have also proposed that the occurrence of PCOS is related to DNA methylation, X-chromosome inactivation, and histone modification (5). With the advancements in the study of intestinal flora in modern medicine, it has been discovered that intestinal flora is closely related to the pathogenesis of PCOS. PCOS patients often exhibit intestinal dysbiosis, which can influence the pathogenesis of PCOS through multiple mechanisms (6).

At present, the treatment of PCOS mainly adopts methods such as lifestyle adjustment and drug therapy. However, these methods have disadvantages such as side effects, low long-term medication compliance of patients, poor curative effects, and the existence of contraindications in some cases. Traditional Chinese medicine (TCM) has accumulated rich experience in the treatment of PCOS through long-term practice and exploration. TCM theory emphasizes the concept of holism, that is, all parts of the human body are interrelated and influence each other. TCM treatment of PCOS has the characteristics of multi-target and overall regulation. Multi-target treatment means that TCM intervenes in multiple symptoms and pathological changes simultaneously. Additionally, TCM has unique potential to regulate intestinal flora, which may be an important mechanism for treating PCOS. In recent years, many clinical studies or animal experiments have attempted to treat PCOS by regulating the intestinal flora with Chinese medicine and have made certain progress. Therefore, gut microbiota and their metabolites may be a crucial therapeutic target for TCM in treating PCOS.

This review conducted a systematic search across major databases, including PubMed, Web of Science, China National Knowledge Infrastructure (CNKI), and Wanfang Data, for articles published in the last decade. The search utilized key terms such as “polycystic ovary syndrome” or “PCOS”, “Traditional Chinese Medicine” or “TCM”, “gut microbiota” or “intestinal flora”, and “mechanism”. The focus was on original clinical and preclinical research articles, as well as review articles that explored the role of gut microbiota in the pathophysiology of PCOS and the modulation of microbiota through TCM in managing PCOS. Studies were selected based on their reporting of mechanistic insights related to hormonal, metabolic, or inflammatory pathways. This selection process involved a two-stage screening, starting with a review of titles and abstracts, followed by a full-text evaluation of potentially eligible studies. The aim is to systematically delineate the tripartite interactions among TCM, PCOS, and gut microbiota, to synthesize evidence on the therapeutic benefits of TCM-driven microbiota modulation in mitigating PCOS pathologies, and to identify and evaluate novel microbiota-targeted modulators derived from TCM compounds, which may offer innovative strategies for clinical management, symptom alleviation, and long-term health improvement in affected women.

## 2 Relationship between intestinal microflora and PCOS

### 2.1 Gut microflora signatures in PCOS

The gut microbiota is essential for maintaining human homeostasis, and an imbalance in this microbiota is associated with polycystic ovary syndrome (PCOS). Research indicates that patients with PCOS exhibit lower levels of α- and β-diversity in their gut microbiota compared to healthy individuals (7, 8). Key microbial changes observed in PCOS include a decrease in beneficial bacteria such as Lactobacillus, Ruminococcus, Clostridium, and Bifidobacterium, along with a reduction in the abundance of Prevotellaceae. Conversely, harmful bacteria like Salmonella pullorum, Bacteroides, and Enterobacteriaceae tend to increase in these patients (9–14). Additionally, some studies have noted an increase in Firmicutes and a decrease in Bacteroidetes in certain models (15). Clinically, dysbiosis in the gut microbiota has been linked to inflammation, insulin resistance, and hyperandrogenism (14, 16). Notably, experiments involving the transplantation of healthy gut microbiota into PCOS rats have shown improvements in ovarian function and hormonal levels, thereby supporting a causal relationship. However, variations across studies highlight that differences in PCOS subtypes, such as obese versus lean individuals, and dietary models, like high-fat diets, can influence these microbial changes (15, 17, 18). This suggests a degree of heterogeneity in the dysbiosis associated with PCOS, as some genera may increase while others decrease.

### 2.2 Mechanism of gut microbiota affecting PCOS

#### 2.2.1 Hyperandrogenism

Hyperandrogenemia (HA) is a key pathological feature of polycystic ovary syndrome (PCOS), leading to various clinical manifestations such as hirsutism, acne, ovulatory dysfunction, and impaired follicular development (19). Additionally, HA plays a role in the progression of PCOS by disrupting ovarian function. The gut microbiota has been shown to influence androgen regulation, particularly testosterone levels. Research indicates that the ratio of Firmicutes to Bacteroidetes correlates with free testosterone levels, and an increase in the abundance of specific bacteria such as Bacteroidaceae, Prevotella, and Parasutterella is associated with elevated testosterone levels (10, 20), with Parasutterella demonstrating a direct positive correlation in PCOS rat models (10, 21). Furthermore, fecal microbiota transplantation studies confirm that gut microbes can directly modulate host androgen levels (11). In terms of PCOS-specific microbial shifts, higher abundances of Anaerococcus and Tyzzerella are linked to increased testosterone levels, while overgrowth of Streptobacillus is associated with pro-inflammatory responses (20, 22, 23). There is a bidirectional interaction between HA and gut microbiota, where androgens can alter the microbiota composition, gut function, and enzyme activity, while dysbiosis can exacerbate HA by further disrupting endocrine and metabolic pathways, thereby worsening the condition of PCOS (7, 24). Early-life effects of HA are also significant, as prenatal HA has been shown to reduce gut microbiota diversity in offspring, which may increase the risk of metabolic diseases in adulthood (25, 26). Clinically, maintaining low androgen levels is crucial for supporting follicular growth, while excess androgens can lead to follicular damage and dermatological symptoms such as acne (27). It is also noteworthy that adrenal HA occurs in 15–45% of PCOS patients, underscoring the heterogeneity of this condition (19).

#### 2.2.2 Insulin resistance

Insulin resistance (IR) is a common endocrine feature in PCOS. Around 50%–70% of PCOS patients present with varying degrees of IR, which is more pronounced in obese patients, who have a higher risk of diabetes compared to normal women (28). The presence of IR not only exacerbates glucose and lipid dysregulation but also contributes to hyperandrogenemia, further complicating the pathology of PCOS. Recent research has highlighted the role of gut microbiota in the development of IR. Notably, shifts in microbial composition, such as a decrease in Prevotella and an increase in Bacteroides, have been associated with IR in PCOS patients (12). The overgrowth of Bacteroides is linked to a reduction in bile acids, including glycine-deoxycholic acid and tauro-ursodeoxycholic acid, as well as impaired secretion of interleukin-22 (IL-22) and disrupted bile acid metabolism (10, 12). In studies involving PCOS rats, an expansion of Prevotella was found to worsen IR and carbohydrate metabolism (29). The mechanisms behind microbial dysbiosis contributing to IR include pathways mediated by lipopolysaccharides (LPS). An increase in LPS-producing bacteria leads to endotoxin leakage, which activates inflammatory pathways such as NF-κB, MAPK, and JNK, resulting in dysfunction of insulin receptor substrates characterized by increased serine phosphorylation and decreased tyrosine phosphorylation, ultimately leading to IR. Additionally, LPS binds to Toll-like receptor 4 (TLR4), further amplifying inflammation and hyperinsulinemia (30). Another critical factor is the depletion of short-chain fatty acids (SCFAs), such as butyrate, which typically enhance insulin sensitivity by regulating gastrointestinal hormones like GLP-1 and PYY and strengthening gut barrier function to reduce endotoxin translocation (14). However, in PCOS, a reduction in beneficial bacteria like Bifidobacterium and Enterococcus faecalis leads to diminished SCFA production, impairing these protective effects and contributing to the overall metabolic dysfunction associated with the condition.

#### 2.2.3 Chronic inflammation

Chronic inflammation is a notable feature in patients with PCOS, marked by low-grade inflammation that manifests through several biological indicators. These patients often show elevated levels of proinflammatory cytokines, both in their ovarian tissue and bloodstream (31, 32). Additionally, there is evidence of leukocyte infiltration, endothelial dysfunction, and metabolic endotoxemia contributing to this inflammatory state. Key microbial metabolites play a significant role in driving this inflammation (33). For instance, short-chain fatty acids (SCFAs) like acetate have been shown to suppress the activation of the NLRP3 inflammasome in PCOS rat models, thereby reducing ovarian inflammation (34, 35). However, other SCFAs, such as butyrate and propionate, which are known to enhance immunomodulation, are often found to be depleted in individuals with PCOS (35). Bile acids (BAs) also exhibit altered profiles in the follicular fluid of PCOS patients, which correlates with hormonal dysregulation, including hyperandrogenemia, and a reduction in IL-22 secretion that leads to impaired immunomodulation (10, 36). Furthermore, lipopolysaccharides (LPS) are implicated in this inflammatory process, disruptions in the gut barrier due to dysbiosis can lead to increased circulating levels of LPS (37). This, in turn, activates the TLR4 receptor, triggering pathways such as NF-κB and MAPK, which promote insulin resistance through mechanisms like IRS-1 serine phosphorylation, as well as hyperandrogenemia and ovarian dysfunction (38, 39). The systemic effects of gut microbiota are also significant, as the interplay between diet and microbiota can exacerbate conditions like insulin resistance and ovarian inflammation, particularly with high-fat diets that increase LPS levels (39, 40). Studies involving microbial transplantation have demonstrated that dysbiosis can directly impact follicular development, oocyte quality, and fertility, linking it to endocrine dysfunction and defects in glycolipid metabolism (10, 41, 42). Notably, there is therapeutic potential in addressing these issues, as supplementation with IL-22 has shown promise in ameliorating insulin resistance and inflammation in models of PCOS (10).

**Figure 1:**
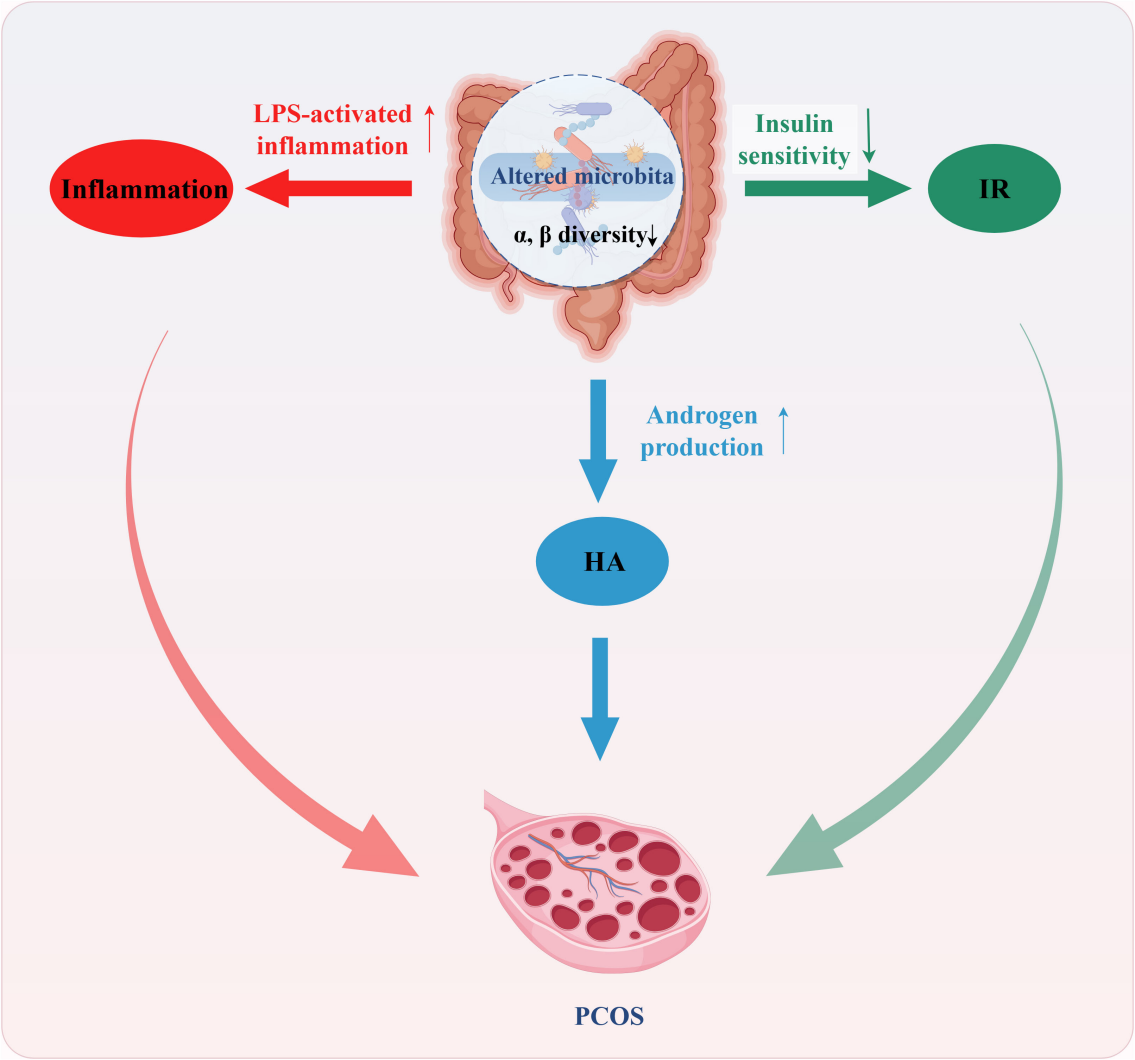
Mechanism of gut microbiota dysbiosis for PCOS. Gut microbiota dysbiosis, characterized by reduced diversity, decreased beneficial bacteria, and increased potentially pathogenic bacteria, may contribute to PCOS through several pathways. The first possible mechanism involves specific bacterial taxa correlating with elevated serum testosterone levels, directly influencing androgen metabolism and leading to increased androgen secretion (Blue). The second possible mechanism is that dysbiosis increases intestinal permeability, allowing bacterial endotoxin lipopolysaccharide (LPS) to translocate into the circulation. This impairs insulin signaling, ultimately resulting in insulin resistance (Green). The third possible mechanism is that LPS translocation triggers systemic inflammation via the TLR4/NF-κB signaling pathway. This elevates pro-inflammatory cytokines, adversely affects follicular development, and reciprocally aggravates hyperandrogenism and insulin resistance (Red).

A summary of the mechanism is shown in **Figure 1**.

## 3 Regulation of TCM on PCOS

PCOS is a prevalent endocrine disorder affecting women of reproductive age, characterized by a range of symptoms including hyperandrogenism, menstrual irregularities, and polycystic ovarian morphology. TCM has been increasingly recognized for its potential in managing PCOS, providing a holistic approach that includes herbal remedies, acupuncture, and lifestyle modifications.

### 3.1 The impact of TCM formulas on PCOS

TCM plays a crucial role in the treatment of PCOS, especially herbal formulas which show unique advantages. On one hand, traditional TCM prescriptions are widely used in the clinical treatment of PCOS patients. A national study in Taiwan showed that more than 20 herbal formulas have been used to treat PCOS patients (43). TCM can regulate the endocrine of PCOS patients by adjusting ovarian hemodynamics, serum hormone levels and menstrual cycles, improving menstrual irregularities, ovulation and pregnancy rates. Moreover, the clinical efficacy of TCM is superior to that of Western medicine treatment (44). A study emphasized that FuFang ZhenZhu TiaoZhi has a positive effect in reducing insulin resistance and improving ovarian function in experimental models of PCOS. It can increase adiponectin levels, showing potential as a therapeutic agent for PCOS (45). Liuwei Dihuang Pills can improve polycystic ovarian changes and reduce follicular atresia (46). Cangfu Daotan Decoction treats PCOS by regulating lipid metabolism, sex hormone secretion and increasing the expression of OATP2B1 and OATP3A1 (47). On the other hand, TCM is widely used and has significant efficacy in regulating intestinal microecology. The comprehensive application of TCM can achieve multi-target and multi-pathway improvement of the dysbiosis of the intestinal flora. Wang et al. (48) found that Bu Shen Hua Zhuo formula can increase the α-diversity of the gut microbiota in PCOS rats, reduce the relative abundance of Firmicutes, increase lactic acid bacteria and short-chain fatty acid-producing bacteria, and improve ovarian morphology. PCOS model was established by orally administering letrozole to rats, which confirmed that Shaoyao-Gancao Decoction significantly reduced the ratio of Firmicutes to Bacteroidetes, decreased the LPS-producing pathogen Proteobacteria, increased the abundance of Butyricicoccus, Coprococcus, Akkermansia, Blautia and Bacteroides, and improved the polycystic symptoms in PCOS rats (49). The traditional Chinese medicine decoction Modified Banxia Xiexin Decoction can regulate the disorder of the intestinal flora in rats, significantly increase the abundance of Verrucomicrobiota, Proteobacteria, Akkermansia and Blautia, reduce the abundance of Clostridium_sensu_stricto_1, and promote follicular development (13). A study on PCOS mice showed that Yulin Tong Bu formula significantly alleviated the dysbiosis of the gut microbiota in PCOS mice, especially increasing the relative abundance of beneficial bacteria such as lactic acid bacteria and short-chain fatty acid-producing bacteria. Metabolites such as ferulic acid and folic acid were negatively correlated with PCOS clinical parameters (50). Jiawei Qi Gong Wan can increase the diversity of the intestinal flora and the number of probiotics in PCOS patients with phlegm-damp constitution, and improve the structure of the intestinal flora (51). In addition, in the treatment of PCOS, TCM methods often use herbal formulas targeting specific syndromes to improve the composition and function of the gut microbiota. Yulin Tong Bu formula can improve PCOS symptoms by correcting glucose metabolism and restoring the balance of the gut microbiota (50); dietary interventions can effectively increase the diversity of the gut microbiota and improve the clinical outcomes of PCOS patients (52). The modulation of gut microbiota by TCM not only aids in the treatment of conditions like PCOS but also offers insights into the underlying mechanisms of herbal efficacy, suggesting that the gut microbiome may serve as a biological indicator for the therapeutic effects of TCM (53).

### 3.2 The effects of single herbs on PCOS

In addition to complex formulas, the efficacy of single herbs and their components in treating PCOS has also been studied. Different monomeric components of traditional Chinese medicine can improve the pathological conditions of PCOS through multiple mechanisms. Coptis chinensis, an important medicinal plant in the Ranunculaceae family, has been found to reverse the pathological damage of ovarian tissue in polycystic ovary syndrome and regulate the mRNA and protein expression levels of MAPK1, CXCL8, IL-6 and IL-1β (54). Cinnamon can improve the menstrual cycle and ovarian size of patients. Short-term supplementation of cinnamon has a beneficial effect on metabolism. For some women with polycystic ovary syndrome, it may be an effective treatment option (55, 56). Wang et al. found that quercetin can improve IR in PCOS rats, show a good therapeutic effect, and restore the estrous cycle of rats (57). Curcumin can significantly reduce body mass index (BMI), fasting blood glucose, insulin levels, and the degree of insulin resistance. In addition, taking curcumin can upregulate the expression of peroxisome proliferator-activated receptor γ (PPAR-γ) gene, peroxisome proliferator-activated receptor γ coactivator 1α (PGC1α) gene, low-density lipoprotein receptor (LDLR) gene, and the activity of glutathione peroxidase (Gpx). Moreover, curcumin can effectively reduce the complications related to oxidative stress in patients with polycystic ovary syndrome and improve insulin sensitivity (58–60). Some other components of traditional Chinese medicine also have positive effects. Berberine is an isoquinoline alkaloid found in plants such as Canadian goldenseal, yellowroot, and California poppy. Relevant studies have shown that it can relieve the progression of PCOS by affecting the production of short-chain fatty acids (SCFAs) by the intestinal flora of PCOS mice (61). In animal models of polycystic ovary syndrome, berberine has neuroprotective and cardiovascular protective effects. Randomized controlled trials (RCTs) have clearly confirmed its lipid-lowering and insulin resistance (IR)-improving effects. It has the same effect as metformin in alleviating insulin resistance, improving glucose and lipid metabolism, and reproductive endocrine status (62). In addition, berberine can improve the changes in Firmicutes and Bacteroidetes at the phylum level and the changes in Romboutsia, Bacteroides, and Clostridium_sensu_stricto_1 at the genus level in the intestinal tract of rats, effectively improving the pathological conditions of PCOS (61). In an open-label, single-group, non-randomized, post-marketing surveillance study, the extract of fenugreek seeds can reduce or dissipate ovarian cysts in patients with polycystic ovary syndrome (PCOS). After treatment, the menstrual cycle of 71% of the patients returned to normal, and 12% of the patients subsequently became pregnant successfully (63). The extract of sage was shown the body mass index and systolic blood pressure of patients with polycystic ovary syndrome and improve the insulin resistance index (64). After taking soy isoflavones for 12 weeks, women with polycystic ovary syndrome showed significant improvements in insulin resistance, hormone levels, total cholesterol, and oxidative stress biomarkers (65). This evidence supports the notion that single herbs can serve as effective adjuncts in the holistic treatment of PCOS, emphasizing the importance of individualized herbal therapy based on specific patient needs.

### 3.3 The influence of acupuncture on PCOS

Acupuncture, a valued aspect of traditional medicine, has been practiced in China for over three thousand years. Over this long history, acupuncture has evolved into multiple treatment approaches, and its importance in treating polycystic ovary syndrome (PCOS) has grown significantly. Both clinical and animal experimental results have shown that acupuncture can effectively regulate the function of the hypothalamic-pituitary-ovarian axis (HPOA) in PCOS patients, improve their metabolic status, and promote ovulation (66). Acupuncture demonstrates unique therapeutic effects by regulating the autonomic nervous system and balancing the activities of the sympathetic and parasympathetic nerves (67, 68). For example, in the case of obese and infertile patients with PCOS, researchers select specific meridians and acupoints for acupuncture treatment, aiming to improve the overall health of these patients (69, 70). Some studies have pointed out that acupuncture treatment can help reduce the LH/FSH ratio, and personalized acupuncture regimens may improve the live birth rates among infertile women (71, 72). However, there are also studies suggesting that acupuncture does not significantly increase the live birth rates (73, 74). In the vast field of modern medicine, the standard treatment methods for obese PCOS mainly include various strategies such as dietary control, exercise-induced weight loss, and behavioral interventions. With its relatively few adverse reactions and the ability to conduct syndrome differentiation and treatment according to individual symptoms, acupuncture shows unique advantages in treating obese PCOS. Current research mainly focuses on the regulation of sex hormone levels by acupuncture and its profound impact on obese PCOS through the ‘brain-gut axis’ mechanism. Research indicates that a high ratio of Firmicutes to Bacteroidetes in the gut microbiota of obese individuals may contribute to obesity, and this dysregulation is viewed as a potential cause of obesity in PCOS patients (24). Acupuncture therapies, such as electroacupuncture and acupoint embedding, can effectively inhibit the accumulation of abdominal fat and regulate glucose and lipid metabolism. This process may be closely related to improvements in the diversity of the gut microbiota and the restoration of its composition and function (75). Acupuncture therapy demonstrates promising potential in regulating gut microbiota, glucose and lipid metabolism, and androgen levels in obese PCOS patients, effectively reducing fat accumulation and enhancing overall health. To further validate these findings, future research should focus on conducting large-scale, long-term randomized controlled trials to establish standardized treatment protocols.

## 4 The mechanism of TCM in regulating PCOS through gut microbiota

**Figure 2:**
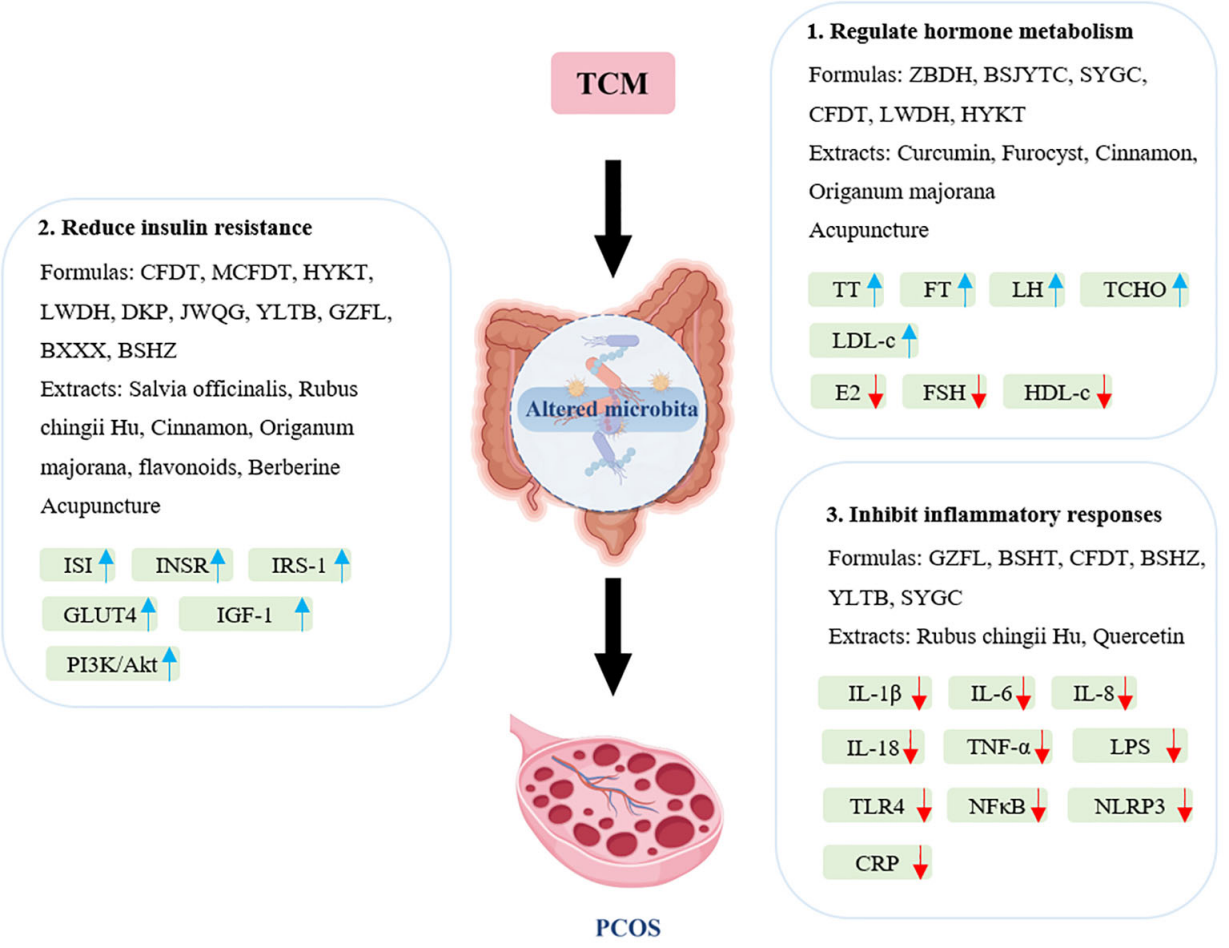
Main mechanisms of traditional Chinese medicines for treating PCOS through modulation of gut microbiota. BSHT, Bushen Huatan Fang; BSJYTC, Bushen Jieyu Tiaochong formula; BSHZ, BuShenHuaZhuo formula; BXXX, Banxia Xiexin decoction; CFDT, Cangfu Daotan decoction; CRP, C-reactive protein; DKP, Dingkun pill; E2, estradiol; FSH, follicle-stimulating hormone; FT, free testosterone; GLUT4, glucose transporter 4; GZFL, Guizhi Fuling Wan; HDL-c, high-density lipoprotein cholesterol; HYKT, Heyan Kuntai capsules; IGF-1, insulin-like growth factor 1; IL, interleukin; INSR, Insulin Receptor; IRS-1, insulin receptor substrate 1; ISI, insulin sensitivity index; JWQG, Jiawei Qigong pills; LDL-c, low-density lipoprotein cholesterol; LH, luteinizing hormone; LPS, lipopolysaccharide; LWDH, Liuwei Dihuang pills; HYKT, Heyan Kuntai Capsules; NFκB, nuclear factor-κB; NLRP3, NLR family pyrin-domain containing protein 3; MCFDT, Modified Cangfu Daotan decoction; SYGC, Shaoyao-Gancao decoction; TCHO, total cholesterol; TLR4, toll-like receptor 4; TNF-α, tumor necrosis factor α; TT, total testosterone; YLTB, YulinTongBu formula; ZBDH, Zhibaidihuang decoction.

Polycystic ovary syndrome (PCOS) is a complex endocrine disorder characterized by hormonal imbalances, insulin resistance, and chronic inflammation, which can lead to various metabolic complications. Traditional Chinese Medicine (TCM) has gained attention as a complementary approach to managing PCOS due to its holistic perspective and multifaceted therapeutic strategies. This section explores the mechanisms by which TCM can improve the symptoms and underlying causes of PCOS, emphasizing hormonal regulation, insulin resistance, and anti-inflammatory effects (**Figure 2**).

### 4.1 Mechanisms of TCM formulas and herbs in regulating PCOS via gut microbiota

#### 4.1.1 Regulate hormone metabolism

PCOS is characterized by specific endocrine features, such as high levels of androgens, increased luteinizing hormone (LH), normal follicle-stimulating hormone (FSH) levels, and a relatively high LH/FSH ratio (76). One of the key mechanisms of TCM in treating PCOS is through the regulation of hormone levels. Relevant studies have shown that various Chinese herbal compound prescriptions have a positive impact in this regard. Modified Zhibaidihuang decoction demonstrates a dose-response relationship in treating hyperandrogenemia in PCOS. After treatment, the concentration of total testosterone in serum decreases, and the acne score is significantly lower than before treatment (77). Research by Pan et al. shows that the Bushen Jieyu Tiaochong formula can enhance abnormal follicular expansion in PCOS rats, while lowering serum levels of free testosterone (FT), LH, and the LH/FSH ratio (78). Shaoyao-Gancao decoction treatment significantly lowers serum testosterone levels in PCOS rats, increases estradiol (E2) and FSH levels, and thus improves hyperandrogenemia in these rats (49, 79). Cangfu Daotan Decoction can reduce the levels of total cholesterol (TCHO), triglycerides (TG), low-density lipoprotein cholesterol (LDL-c), luteinizing hormone, and testosterone in the serum of PCOS rats, and increase the levels of high-density lipoprotein cholesterol (HDL-c), follicle-stimulating hormone, and estradiol in a dose-dependent manner (47). Liuwei Dihuang pills significantly increase the levels of serum FSH, E2, and progesterone in PCOS rats while reducing the levels of LH and testosterone (46). Liang et al. (80) found that Heyan Kuntai capsules can improve the levels of serum LH, LH/FSH, and testosterone in patients with PCOS, reduce levels of total cholesterol, triglycerides, and LDL-c in patients, and increase the level of HDL-c. In addition, some other Chinese herbal components also have a regulatory effect on hormone metabolism. One study has confirmed that Curcumin is associated with a significant reduction in total cholesterol, LDL cholesterol, and the total/HDL cholesterol ratio, as well as a significant increase in the HDL cholesterol level (60). The extract of Trigonella foenum-graecum seed (Furocyst) has been found to increase the levels of LH and FSH in the serum of patients with PCOS (63). Cinnamon can improve the levels of total cholesterol (TC) and LDL in patients (55). Another study found that Origanum majorana can significantly reduce the level of adrenal androgens (81). TCM uses hormonal adjustments to relieve PCOS symptoms and tackle the disorder’s root causes, offering a more holistic treatment approach.

#### 4.1.2 Reduce insulin resistance

Insulin resistance (IR) is a key characteristic of PCOS. It regulates various mediators and pathways, playing a crucial role in the development and progression of PCOS. It also leads to metabolic dysfunction and an increased risk of type 2 diabetes. The potential of TCM to enhance insulin sensitivity through various mechanisms is well recognized. Several Chinese herbal compound prescriptions have demonstrated significant effectiveness in improving insulin resistance in both PCOS patients and rats. Wang et al. (82) confirmed that the Chinese herbal compound Cangfu Daotan decoction can improve IR in PCOS rats, restore serum hormone levels, and reduce ovarian morphological damage in PCOS rats. Modified Cangfu Daotan decoction can play a role in improving ovarian function in PCOS-IR rats by upregulating the expression of INSR/IRS-1/GLUT4 in the insulin signaling pathway in an inflammatory environment (83). Heyan Kuntai capsules can improve metabolic disorders in PCOS patients, reduce body mass index and waist-to-hip ratio, decrease fasting and 2-hour postprandial blood glucose levels, increase the insulin sensitivity index (ISI) and decrease sex hormone levels (80). Qiu et al. (46) found that treatment with Liuwei Dihuang pills normalized the insulin sensitivity index, significantly improved the structure of polycystic ovaries, and reduced insulin resistance by upregulating Cyp19a1 and activating the PI3K/Akt signaling pathway. In PCOS patients, Dingkun pill (DKP) or the combination of DKP and Diane-35 can lead to a decrease in the homeostasis model assessment of insulin resistance and an increase in the quantitative insulin sensitivity check index (84). Jiawei Qigong pills can improve the insulin resistance state, regulate endocrine metabolism, and improve overall symptoms by improving the intestinal flora structure in PCOS patients with phlegm-damp constitution (51). Treatment with YulinTongBu formula has improved symptoms such as delayed blood glucose clearance, decreased insulin sensitivity, disorders of glucose and lipid metabolism, and hormonal imbalance in PCOS mice (50). In addition, some plant extracts also have a positive effect on improving insulin resistance in PCOS patients. The extract of Salvia officinalis can significantly reduce insulin levels and the homeostasis model assessment of insulin resistance in PCOS patients, and significantly increase the quantitative insulin sensitivity check index, thus improving insulin resistance markers (64). Cinnamon can significantly reduce fasting insulin and insulin resistance in women with PCOS (55). Origanum majorana can reduce insulin levels and the homeostasis model insulin resistance index, improve insulin sensitivity in women with polycystic ovary syndrome (81). Treatment with Rubus chingii Hu has improved hormonal imbalance and IR in PCOS model rats and ameliorated ovarian pathological conditions (85). Furthermore, electroacupuncture treatment can improve ovarian IR in PCOS patients by upregulating the IRS-1/PI3K/GLUT4 signaling pathway (86). In the PCOS-IR rat model, total flavonoids can decrease insulin levels, increase IRS-1 and p-IRS-1 levels, and improve the histopathological changes in the ovaries and pancreas (87).

Multiple studies indicate that an imbalance in gut microbiota plays a role in both the onset and persistence of insulin resistance (88). Zhu et al. (89) induced PCOS model rats and found that Guizhi Fuling Wan can significantly increase the relative abundance of Alloprevotella and reduce the relative abundance of RuminococcaceaeUCG-003 and LachnospiraceaeUCG-008, thereby alleviating IR in PCOS rats. Zhao et al. (13) administered Modified Banxia Xiexin decoction to PCOS-IR model rats and discovered that it can reduce insulin resistance by lowering the relative abundance of the key pathogenic bacterium Clostridium sensu stricto 1. The Bushen Huazhuo formula (48) significantly increases the α-diversity of gut microbiota in PCOS model rats. It decreases the abundance of Firmicutes while increasing Lactobacillus and gut bacteria that metabolize short-chain fatty acids (SCFAs), leading to improved insulin resistance. Shen et al. (61) found that Berberine, an isoquinoline alkaloid from the Chinese herbal medicine Coptis chinensis, alters the relative abundance of Firmicutes and Bacteroidetes, enhances relevant metabolites, and lowers fasting blood glucose and insulin levels, as well as the insulin resistance index. TCM addresses insulin resistance, which aids in managing weight and metabolic health in patients with polycystic ovary syndrome. Additionally, it reduces the risk of long-term complications associated with this condition.

#### 4.1.3 Inhibit inflammatory responses

Chronic inflammation is a major factor in the pathophysiology of PCOS, contributing to insulin resistance and hormonal imbalance. Multiple studies have shown that various Chinese herbal formulas significantly regulate chronic inflammation in PCOS. Zhu et al. (89) found that Guizhi Fuling Wan significantly reduces concentrations of inflammatory factors like interleukin-6 (IL-6), tumor necrosis factor-α (TNF-α), and high-sensitivity C-reactive protein. This reduction inhibits the inflammatory response in PCOS-IR model rats. This effect is linked to changes in the relative abundance of gut microbiota, which influences inflammation levels. Among them, the relative abundance of Alloprevotella is negatively correlated with the levels of inflammatory markers IL-6 and high-sensitivity C-reactive protein, while the relative abundances of Ruminococcaceae UCG-003 and Lachnospiraceae UCG-008 are positively correlated with the levels of IL-6 and high-sensitivity C-reactive protein. Oxidative stress and inflammation are closely related to the occurrence of PCOS. Lu Chen et al. have confirmed that Bushen Huatan Fang can reduce the inflammatory response and oxidative stress in PCOS (90). Cangfu Daotan decoction can regulate lipid metabolism, sex hormone secretion and inflammatory response, and reduce the serum levels of IL-1β, IL-6 and TNF-α (47), also can improve the inflammatory microenvironment and regulate follicular development by regulating the IGF-1-PI3K/Akt-Bax/Bcl-2 signaling pathway (82). Both BuShenHuaZhuo formula and YulinTongBu formula can reduce the levels of lipopolysaccharide (LPS) and inflammatory cytokines such as TNF-α, IL-6 and IL-8 in the serum of PCOS rats, and inhibit the inflammatory response mediated by the nuclear factor-κB (NF-κB) signaling pathway (48, 50). Shaoyao-Gancao decoction treatment can not only effectively reduce the phosphorylation of NF-κB p65, increase the expression of IκB, and decrease the levels of inflammatory factors such as TNF-α, IL-1β, IL-6 and IL-18 in the serum and ovarian tissues of PCOS rats (79), but also enhance the expression of tight junction proteins (occludin and claudin1) and inhibit the expression of key genes and proteins in the TLR4/NF-κB signaling pathway (49). In addition, the treatment with the Chinese herbal monomer Rubus chingii Hu can inhibit the activation of the TXNIP/NLRP3 inflammasome in the ovarian tissue of PCOS rats and improve the polycystic development of the ovaries (85). Quercetin can reduce the levels of IL-1b, IL-6, TNF-α and NF-κB, improve the inflammatory microenvironment of ovarian tissue in PCOS rat models, and inhibit the TLR/NF-κB signaling pathway (57). The anti-inflammatory effects of Chinese herbal medicine can relieve PCOS symptoms and prevent the progression of related metabolic disorders, thus improving patients’ quality of life.

A summary of this section is shown in **Tables 1**, **2**.

**Table 1:** Clinical trial of treating PCOS with TCM.

Reference	Intervention	Patients	Duration of study	Results	Mechanism
(51)	Jiawei Qi Gong Wan	60	2 months	increase the number of intestinal probiotics and improve the state of insulin resistance	reduces insulin resistance
(55)	cinnamon powder	66	12 weeks	improve the levels of TC and LDL-c	reduces insulin resistance
(59)	Curcumin	60	6 weeks	increase insulin sensitivity check index	reduces insulin resistance
(60)	Curcumin	60	12 weeks	reduce TC, LDL-c; increase HDL-c and insulin sensitivity	regulate hormone metabolism; reduces insulin resistance
(63)	Trigonella foenum-graecum seed extract	50	90 days	increase the levels of LH and FSH	regulate hormone metabolism
(64)	Salvia officinalis extract	60	8 weeks	reduced insulin levels and HOMA-IR	reduces insulin resistance
(65)	Soy Isoflavones	70	12 weeks	reduce HOMA-IR, TC, and free androgen index; increase insulin sensitivity check index	regulate hormone metabolism; reduces insulin resistance
(77)	Zhibaidihuang Decoction	90	4 months	decrease in serum testosterone concentration; Reduction in acne scores	regulate hormone metabolism
(80)	Kuntai capsules	100	6 months	improve the levels of serum LH, LH/FSH, and testosterone; increase insulin sensitivity index	regulate hormone metabolism; reduces insulin resistance
(81)	Origanum majorana	25	1 months	reduce the level of adrenal androgens; improve insulin sensitivity	regulate hormone metabolism; reduces insulin resistance
(84)	Dingkun pill	117	3 months	increase insulin sensitivity check index	reduces insulin resistance

HOMA-IR, homeostasis model assessment of insulin resistance; TC, total cholesterol; LDL-c, low-density lipoprotein cholesterol; HDL-c, high-density lipoprotein cholesterol.

**Table 2:** Animal experiment of treating PCOS with TCM through gut microbiota.

Reference	Intervention	Animals	Model	Results	Mechanism
(13)	Banxia Xiexin Decoction	Rats	Letrozole plus HFD	increase the abundance of Verrucomicrobiota, Proteobacteria, Akkermansia and Blautia; reduce the abundance of Clostridium_sensu_stricto_1, and promote follicular development	reduces insulin resistance
(48)	Bu Shen Hua Zhuo formula	Rats	Letrozole	increase the α-diversity of the gut microbiota; reduce the relative abundance of Firmicutes; increase lactic acid bacteria and short-chain fatty acid-producing bacteria	regulate hormone metabolism; inhibit inflammatory responses
(49)	Shaoyao Gancao Decoction	Rats	Letrozole	reduced the ratio of Firmicutes to Bacteroidetes; decreased the LPS-producing pathogen Proteobacteria; increased the abundance of Butyricicoccus, Coprococcus, Akkermansia, Blautia and Bacteroides	regulate hormone metabolism; inhibit inflammatory responses
(50)	Yulin Tong Bu foumula	Mice	DEHA plus HFD	increasing the relative abundance of beneficial bacteria such as lactic acid bacteria and short-chain fatty acid-producing bacteria	inhibit inflammatory responses
(61)	Berberine	Rats	DHEA	improve the changes in Firmicutes and Bacteroidetes at the phylum level and the changes in Romboutsia, Bacteroides, and Clostridium_sensu_stricto_1 at the genus level	reduces insulin resistance
(78)	Bushen Jieyu Tiaochong Formula	Rats	Letrozole plus CUMS	decreases the abundance of Firmicutes, increasing Lactobacillus and gut bacteria	regulate hormone metabolism
(89)	Guizhi Fuling Wan	Rats	Letrozole plus HFD	increase the relative abundance of Alloprevotella and reduce the relative abundance of RuminococcaceaeUCG-003 and LachnospiraceaeUCG-008	reduces insulin resistance; inhibit inflammatory responses

DEHA, dehydroepiandrosterone; HFD, high fat diet; CUMS, Chronic unpredictable mild stress.

### 4.2 Mechanisms of acupuncture in regulating PCOS via gut microbiota

Acupuncture, a fundamental practice in Traditional Chinese Medicine with a history spanning thousands of years, offers therapeutic benefits for polycystic ovary syndrome (PCOS) through mechanisms that differ from those of orally administered herbal remedies. While herbal treatments interact directly with the gastrointestinal tract and its microbiota, acupuncture primarily works by stimulating specific acupoints on the body surface, which sends afferent neural signals to the central nervous system (CNS) and triggers a series of neuroendocrine responses. A crucial pathway for the systemic effects of acupuncture, including its impact on gut microbiota and PCOS-related issues, is the brain-gut axis (91). The neuroendocrine signals activated by acupuncture, particularly through the vagus nerve, can significantly affect gut physiology and alter the composition of gut microbiota via this brain-gut connection. Research involving PCOS models has shown that acupuncture can effectively modify the gut microbial ecosystem. For instance, a randomized controlled trial found that acupuncture combined with clomiphene reduced the luteinizing hormone to follicle-stimulating hormone ratio and improved insulin resistance in obese PCOS patients, suggesting a potential link to gut microbiota (92). Furthermore, moxibustion has been shown to influence gut microbiota by increasing the levels of beneficial bacteria such as UCG-005 and Turicibacter while decreasing Desulfovibrio levels. It also helps alleviate PCOS symptoms by lowering fasting blood glucose, testosterone, and insulin levels (93). Additionally, electroacupuncture (EA) has been found to normalize serum levels of dihydrotestosterone (DHT) and progesterone, enhance glucose tolerance, and modify gut microbiota composition by increasing the abundance of the phylum Tenericutes and the genus Prevotella_9, thereby improving metabolic dysfunction and reproductive function in a PCOS-like rat model (94).

## 5 Discussion

Overall, substantial evidence links gut microbiota dysbiosis to the pathogenesis of PCOS. TCM, which includes herbal formulas such as Bu Shen Hua Zhuo formula, Shaoyao-Gancao Decoction, and Modified Banxia Xiexin Decoction, as well as individual herbs like Berberine and Curcumin, has shown significant therapeutic potential in managing PCOS. The modulation of gut microbiota is increasingly recognized as a key mechanistic pathway through which TCM exerts its beneficial effects, contributing to the restoration of hormonal balance, enhancement of insulin sensitivity, and reduction of chronic inflammation.

While modern medications, such as combined oral contraceptives (COCs) for hyperandrogenism and metformin for insulin resistance, effectively target specific symptoms, they often come with limitations, including side effects like gastrointestinal issues from metformin and COCs (95), as well as an incomplete understanding of the complexities of PCOS, particularly concerning gut dysbiosis. In contrast, TCM presents a holistic alternative that addresses core PCOS pathologies including hyperandrogenism, insulin resistance, and inflammation, primarily via gut microbiota modulation. Notable distinctions include mechanistic divergence, where metformin activates AMPK peripherally, while TCM formulas, such as Modified Banxia Xiexin Decoction, correct dysbiosis by reducing Clostridium_sensu_stricto_1 and boosting SCFA producers, thereby dampening inflammation and endotoxemia. Additionally, while COCs suppress ovarian androgens, TCM, exemplified by Modified Zhibaidihuang Decoction, normalizes gut taxa associated with androgen metabolism, including Parasutterella and Bacteroides. The synergistic potential of TCM lies in its ability to combat dysbiosis and inflammation, which may enhance the efficacy of conventional drugs, such as by mitigating the gastrointestinal effects of metformin through gut barrier repair, allowing for lower drug doses.

However, current research in this area faces several challenges. Many clinical trials and animal studies suffer from limited sample sizes, restricting statistical power and the generalizability of findings. The validity of existing animal models in accurately representing the complex pathophysiology of human PCOS is also a point of contention, which hampers their applicability to real-world scenarios. Furthermore, inconsistencies in the sourcing and preparation of TCM formulations pose difficulties for mechanistic studies and reproducibility. Often, there is insufficient detailed characterization of microbial changes, and a lack of comprehensive metabolomic profiling that links TCM interventions with shifts in gut microbiota and host outcomes. To fully realize the therapeutic potential of TCM via gut microbiota modulation, it is essential to align TCM practices with modern clinical standards. This alignment necessitates rigorous standardization and quality control of TCM products, employing advanced analytical techniques such as chromatography. Although there is evidence indicating that specific TCM compound formulas or individual herbs can influence gut microbiota, the precise active components and their molecular targets remain unidentified.

While there is evidence that specific TCM compound formulas or individual herbs can influence gut microbiota, the exact active components and their molecular targets remain unclear. Therefore, future research should focus on identifying these specific active components within TCM formulations that are responsible for modulating gut microbiota and alleviating symptoms of PCOS, utilizing modern biotechnologies like metabolomics and proteomics for a more thorough investigation. Additionally, exploring integrative approaches that combine TCM with conventional therapies is necessary to examine potential synergistic effects. Furthermore, the development of PCOS is influenced by various factors, including endocrine, metabolic, genetic, and environmental influences, and the mechanisms of interaction among these factors warrant further exploration.
